# Mitochondrial respiration and nucleotide profiling in human left internal mammary artery from CABG: a novel real-time *ex vivo* approach

**DOI:** 10.3389/abp.2026.16877

**Published:** 2026-07-27

**Authors:** Alicja Braczko, Andrzej Łoś, Marta Piotrowska, Ada Kawecka, Iga Walczak, Mikołaj Krysiak, Marcin Hellmann, Maciej Brzeziński, Ryszard T. Smoleński, Barbara Kutryb-Zając

**Affiliations:** 1 Department of Biochemistry, Medical University of Gdańsk, Gdańsk, Poland; 2 Centre of Experimental Cardioonocology, Medical University of Gdańsk, Gdańsk, Poland; 3 Department of Cardiac and Vascular Surgery, Medical University of Gdańsk, Gdańsk, Poland; 4 Department of Histology, Medical University of Gdańsk, Gdańsk, Poland; 5 Department of Cardiac Diagnostics, Medical University of Gdańsk, Gdańsk, Poland

**Keywords:** CABG, *ex vivo* model, LIMA, mitochondrial respiration, seahorse XF flex 3D

## Abstract

Mitochondrial dysfunction plays a critical role in the pathogenesis of cardiovascular and metabolic diseases. However, direct assessment of mitochondrial respiration in human vascular tissue remains technically challenging. In this study, we present an *ex vivo* approach for real-time analysis of mitochondrial respiration in human left internal mammary artery (LIMA) grafts obtained during coronary artery bypass grafting (CABG). LIMA segment was collected intraoperatively and processed for bioenergetic assessment using the Seahorse XF Flex 3D Analyzer. Mitochondrial respiration was evaluated using the Mito Stress Test, enabling real-time measurement of oxygen consumption rate (OCR). In parallel, tissue nucleotide levels were quantified using high-performance liquid chromatography (HPLC), allowing complementary assessment of cellular energy status and redox balance. We demonstrate the feasibility of measuring mitochondrial respiration in intact human arterial tissue *ex vivo*. To our knowledge, this is the first application of the Seahorse XF Flex 3D platform for real-time bioenergetic analysis in intact human vascular tissue. Combined analysis of OCR and nucleotide levels enabled integrated assessment of vascular bioenergetic status. The applied protocol enabled reliable assessment of key bioenergetic parameters, including basal and maximal respiration. This study establishes a novel proof-of-concept workflow for *ex vivo* bioenergetic profiling of human vascular grafts, providing a platform for future investigations of vascular metabolism in cardiovascular and metabolic disorders.

## Introduction

Mitochondria play a central role in vascular homeostasis, extending beyond ATP production to regulate redox signalling, calcium handling and cellular stress responses (Xu et al., 2025). In endothelial cells, mitochondria function primarily as signalling organelles rather than major energy producers, critically modulating vascular tone, inflammation and adaptation to metabolic stress (Xie et al., 2010; Groschner et al., 2012).

Accumulating evidence indicates that mitochondrial dysfunction contributes to the pathogenesis of cardiovascular and metabolic disorders. Impaired mitochondrial function leads to excessive reactive oxygen species (ROS) production, endothelial activation and reduced nitric oxide bioavailability, promoting vascular stiffness, inflammation and atherosclerosis (Walczak et al., 2026). These processes are particularly relevant in patients undergoing coronary artery bypass grafting (CABG), where the functional integrity of vascular grafts is essential for long-term outcomes (Emmert et al., 2024).

The left internal mammary artery (LIMA) is considered the gold standard conduit in CABG due to its superior long-term patency and resistance to atherosclerosis (Shadrin et al., 2023). Despite its clinical importance, the bioenergetic profile of LIMA remains poorly characterized, largely due to technical limitations in assessing mitochondrial function in intact human vascular tissue.

Recent advances in extracellular flux analysis, such as the Seahorse XF platform, have enabled real-time assessment of mitochondrial respiration in intact cells and isolated mitochondria (Brand and Nicholls, 2011; Divakaruni et al., 2014). However, their application to intact human vascular tissues remains limited.

In this study, we aimed to establish a novel *ex vivo* approach for real-time bioenergetic analysis of intact human vascular tissue using the Seahorse XF Flex 3D Analyzer. Using LIMA grafts obtained during CABG, we demonstrate the feasibility of measuring mitochondrial respiration in structurally preserved human arterial segments. This proof-of-concept study introduces, to our knowledge, the first application of Seahorse XF Flex 3D technology to intact human vascular tissue and provides a new platform for investigating vascular mitochondrial function in clinically relevant settings.

## Materials and methods

### Patient characteristics

A 67-year-old male patient undergoing elective CABG was included in this study. The patient presented with stable two-vessel coronary disease, arterial hypertension and orally treated diabetes mellitus. LIMA was harvested during a standard surgical technique with the sternotomy access and used as a graft to the left anterior descending artery. A surplus/residual segment of LIMA was collected for *ex vivo* analysis. All procedures were conducted in accordance with the Declaration of Helsinki and approved by the local bioethics committee at the Medical University of Gdańsk, Poland (No. KB/259/2025). Written informed consent was obtained from the patient prior to inclusion in the study.

### Tissue processing

**FIGURE 1:**
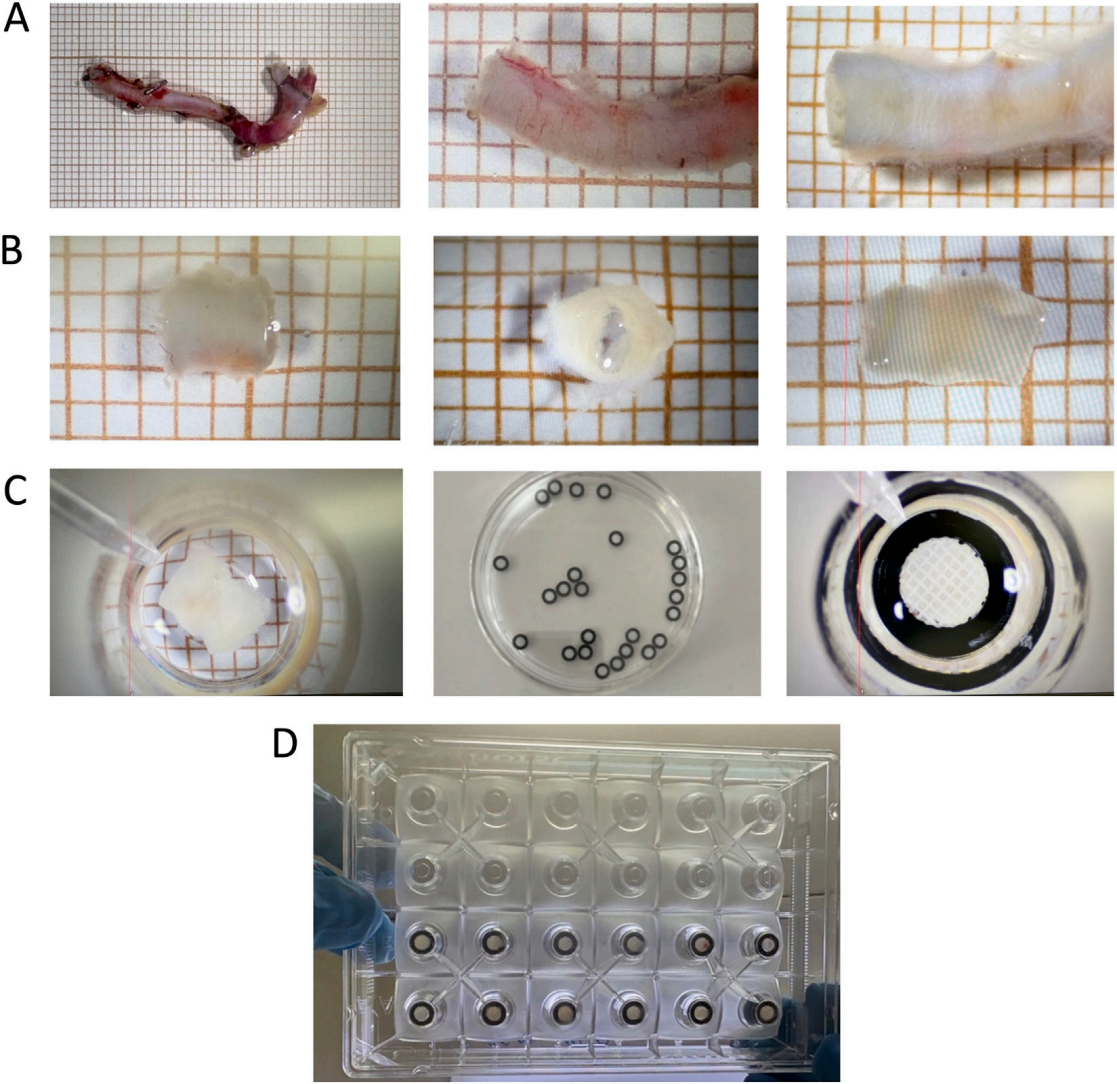
Stepwise preparation of human LIMA samples for *ex vivo* bioenergetic analysis using the Seahorse XF Flex 3D platform. **(A)** Harvesting and preparation of the left internal mammary artery (LIMA) segment obtained during coronary artery bypass grafting (CABG), including removal of surrounding connective tissue and longitudinal opening of the vessel. **(B)** Preparation of flat vascular tissue segments suitable for Seahorse analysis. **(C)** Placement of the tissue onto the Seahorse XF capture microplate, positioning of the islet capture screen, and immobilization of the sample within the measurement well. **(D)** Final arrangement of prepared LIMA samples in the Seahorse XF Flex 3D Capture Microplate before mitochondrial respiration measurements.

LIMA segment was obtained intraoperatively during CABG procedure. Immediately after harvesting, the vessel was placed in Agilent Seahorse XF DMEM Medium (pH 7.4) and transported to the laboratory. The vessel was cleaned of surrounding tissue and sectioned into small fragments suitable for analysis. The vascular rings were then cut longitudinally and gently flattened to obtain planar tissue segments. The samples with the luminal surface facing upwards were immobilized and placed onto Seahorse XF capture microplates equipped with islet capture screens, ensuring that the endothelial layer was exposed to the measurement chamber (Figure 1).

### Histological staining

LIMA sections were fixed in 4% buffered formaldehyde and embedded in paraffin. The embedded artery fragments were cut into 6 µm-thick cross-sections and placed on microscope slides. LIMA sections were deparaffinized with xylene and rehydrated in a descending alcohol series to water. Slides were stained with hematoxylin and eosin (HE) to assess vascular structure and confirm the presence of an endothelial lining.

### Seahorse XF flex mito stress assay

The mitochondrial function of vascular tissue was assessed using the Agilent Seahorse XF Flex analyzer with the Seahorse XF Flex 3D Capture Microplate-L. LIMA segments were cut into 2–3 mm long fragments (n = 3) to fit the microplate wells. One day prior to the assay, the sensor cartridge was hydrated with 1 mL of Seahorse XF calibrant solution per well and incubated at 37 °C in a non-CO_2_ incubator overnight. On the day of the experiment, vessel segments were loaded into wells containing assay medium, and the capture screen was positioned to secure the tissue. The mitochondrial stress test was conducted by sequential injection of compounds in the following order: oligomycin A at a final well concentration of 20 μM, FCCP at 15 μM, and a mixture of rotenone and antimycin A each at 10 µM. Oxygen consumption rates were recorded to measure mitochondrial respiration parameters including basal respiration, ATP-linked respiration, maximal respiration, proton leak, and non-mitochondrial respiration in the LIMA segments. Total protein content in each sample was determined using the Bradford assay according to the manufacturer’s instructions. Oxygen consumption rate (OCR) values were normalized to protein content and are presented as pmol O_2_/min/mg protein.

### Adenine nucleotide and nicotinamide adenine dinucleotide measurements

Tissue nucleotide concentrations were determined using high-performance liquid chromatography (HPLC) as described earlier (Smolenski et al., 1990; Karaś et al., 2024). Briefly, LIMA sections (n = 3) were snap-frozen and homogenized in ice-cold perchloric acid to extract nucleotides. After neutralization, samples were centrifuged, and the supernatant was subjected to HPLC analysis. Adenosine triphosphate (ATP), diphosphate (ADP) and monophosphate (AMP) as well as guanosine triphosphate (GTP) and diphosphate (GDP) and oxidized (NAD^+^) and reduced (NADH) nicotinamide adenine dinucleotide were measured. As adenosine 5′-diphosphoribose (ADPR) is the major product formed by acidic cleavage of NADH during the tissue extraction, the measured ADPR level reflected NADH concentration. Quantification was performed using external standards, and results were normalized to total protein content in sample.

## Results

**FIGURE 2:**
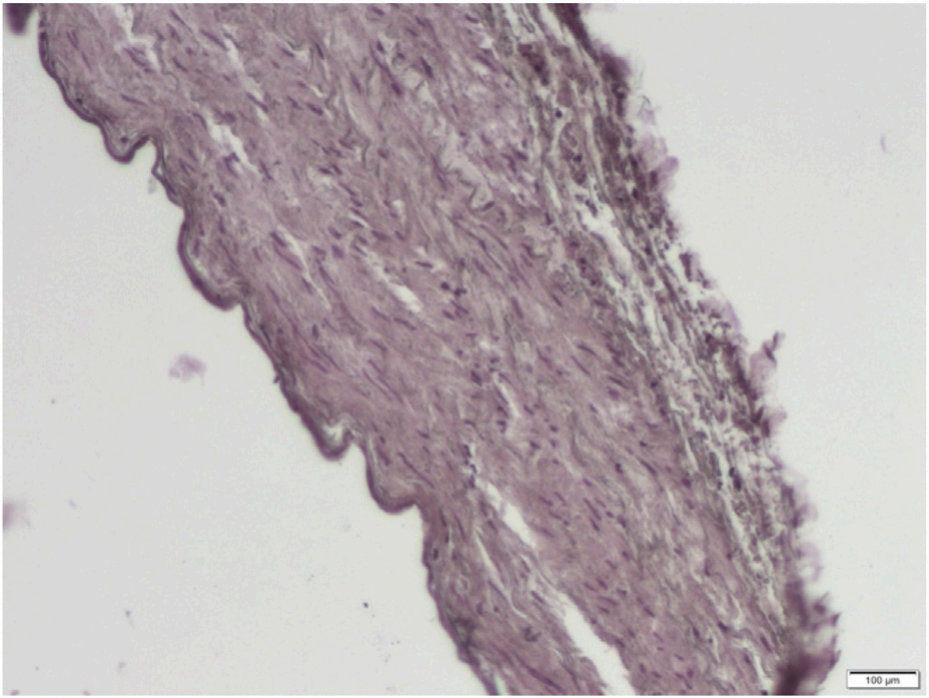
Histological validation of LIMA structure. Representative histological images of hematoxylin and eosin (HE) staining of LIMA segments obtained during CABG (×400 magnification).

We successfully established an *ex vivo* protocol for measuring mitochondrial respiration in human LIMA tissue using the Seahorse XF Flex Analyzer. Histological analysis confirmed preservation of vascular structure in prepared LIMA segments (Figure 2).

**FIGURE 3:**
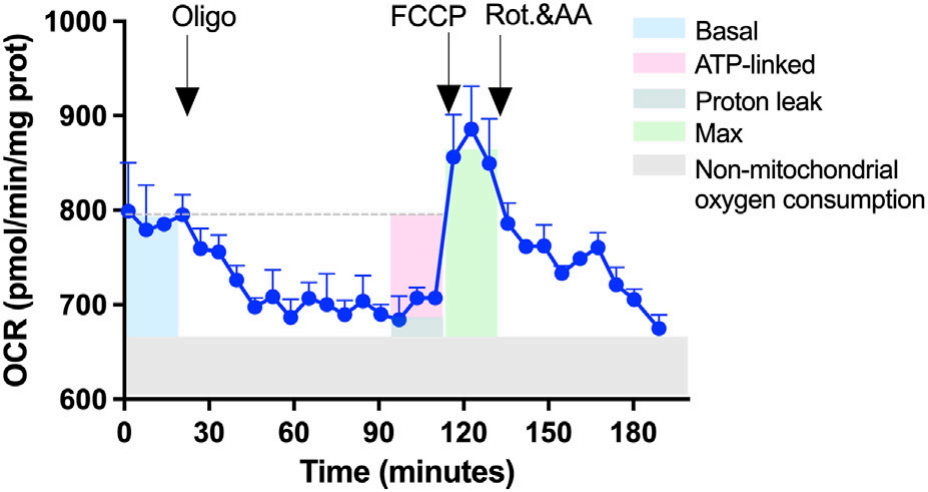
Real-time mitochondrial respiration in human LIMA assessed by the Seahorse XF Flex Mito Stress Test. Oxygen consumption rate (OCR) was measured in *ex vivo* LIMA segments (n = 3) obtained during CABG. Sequential injections of oligomycin (Oligo), trifluoromethoxy carbonylcyanide phenylhydrazone (FCCP) and rotenone/antimycin A (Rot.&AA) were used to evaluate the parameters, including basal respiration (basal), ATP-linked respiration (ATP-linked), maximal respiration (max), proton leak and non-mitochondrial respiration.

Real-time OCR measurements demonstrated that mitochondrial respiration can be reliably assessed in intact human arterial tissue using the Seahorse XF Flex 3D platform (Figure 3). The applied protocol enabled reproducible measurement of key bioenergetic parameters, including basal respiration, ATP-linked respiration and maximal respiratory capacity.

**FIGURE 4:**
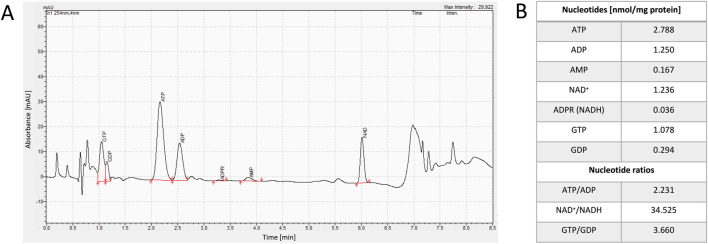
Nucleotide profiling in human LIMA tissue. **(A)** Representative chromatogram of high-performance liquid chromatography of nucleotide extracts obtained from *ex vivo* LIMA segments collected during CABG. **(B)** Peaks corresponding to adenine and guanine nucleotides, as well as oxidized (NAD^+^) and reduced (NADH) nicotinamide adenine dinucleotide. NADH is presented as an equivalent of adenosine 5′-diphosphoribose (ADPR), which is the major product of acidic cleavage during the tissue extraction. All metabolites are identified based on retention times of external standards. Quantitative analysis of nucleotide concentrations in LIMA tissue is presented in the table. Levels of adenosine triphosphate (ATP), adenosine diphosphate (ADP), adenosine monophosphate (AMP), NAD^+^ and NADH, guanosine triphosphate (GTP) and guanosine diphosphate (GDP) were measured and normalized to protein content.

In parallel, HPLC analysis confirmed the presence of measurable levels of nucleotides in LIMA tissue (Figure 4). Nucleotide profiling demonstrated preserved energy status of the analyzed samples, supporting the functional integrity of the vascular tissue under *ex vivo* conditions. Combined assessment of mitochondrial respiration and nucleotide concentrations provides complementary insight into vascular bioenergetics at both functional and metabolic levels.

## Discussion

In this study, we demonstrate the feasibility of real-time *ex vivo* assessment of mitochondrial respiration in human LIMA grafts using the Seahorse XF Flex 3D Analyzer. To our knowledge, this is the first application of the Seahorse XF Flex 3D platform for bioenergetic analysis in intact human vascular tissue.

To date, the assessment of mitochondrial function in the vascular system has been primarily performed in isolated endothelial or vascular smooth muscle cells as well as intact vessels from experimental animal models (Karaś et al., 2024; Yu et al., 2017; Fang et al., 2025). These approaches have provided important insights into vascular bioenergetics, but they do not fully capture the structural and cellular complexity of intact human vascular tissue.

The application of the Seahorse XF Flex 3D platform to structurally preserved human arterial segments therefore represents a methodological advancement, enabling bioenergetic measurements under conditions that more closely resemble the *in vivo* environment. The ability to measure oxygen consumption rate in real time in intact human vessels provides a novel tool for studying vascular bioenergetics in clinically relevant settings.

In this study, we complemented functional assessment of mitochondrial respiration with biochemical quantification of nucleotide levels using HPLC. While extracellular flux analysis provides dynamic information on mitochondrial activity, nucleotide profiling reflects the energetic state of the tissue. Importantly, the integration of these approaches allows simultaneous assessment of functional mitochondrial activity, cellular energy and redox status for a more comprehensive evaluation of vascular bioenergetics.

The left internal mammary artery, widely used in CABG due to its superior long-term patency, represents an ideal model for translational studies of vascular function (Karthi et al., 2006). Despite its clinical importance, its bioenergetic profile has been poorly characterized. The methodological approach presented here provides a new opportunity to investigate metabolic features of clinically relevant human vascular grafts. Thus, the integration of mitochondrial respiration measurements with nucleotide analysis may be particularly valuable in future studies aimed at identifying metabolic alterations associated with vascular dysfunction or patient comorbidities.

Importantly, the ability to assess mitochondrial respiration in human LIMA tissue may have potential clinical implications for evaluating graft quality. Although LIMA is widely regarded as the gold standard conduit in CABG, variability in endothelial function and metabolic status between patients may influence graft performance and long-term patency. Clinical studies have shown that both biochemical and patient-specific factors significantly affect short- and long-term outcomes following cardiac surgery (INFLACOR study) (Kowal et al., 2018). However, current intraoperative assessment of graft quality is largely limited to anatomical and flow-based measurements, which do not provide insight into the metabolic condition of the vessel (Arslanhan et al., 2026). *Ex vivo* bioenergetic profiling using this approach may therefore offer a complementary functional readout reflecting mitochondrial integrity and cellular viability within the vascular wall. Such an approach may help identify subtle alterations in vascular metabolism associated with patient comorbidities, such as diabetes or oxidative stress. In the future, this methodology could contribute to the development of biomarkers of graft quality and support personalized strategies in coronary artery bypass grafting.

### Limitations

This study represents a proof-of-concept analysis based on a single human LIMA and should therefore be considered preliminary. The findings demonstrate technical feasibility rather than providing generalizable biological conclusions. In addition, variability related to tissue handling, preparation and positioning within the Seahorse platform may influence the measurements and requires further standardization. Future studies involving larger patient cohorts are necessary to validate the robustness, reproducibility, and potential clinical relevance of this approach.

## Data Availability

The original contributions presented in the study are included in the article/supplementary material, further inquiries can be directed to the corresponding author.
